# Awareness, experiences and perceptions of telehealth in a rural Queensland community

**DOI:** 10.1186/s12913-015-1094-7

**Published:** 2015-09-28

**Authors:** Natalie K. Bradford, Liam J. Caffery, Anthony C. Smith

**Affiliations:** The University of Queensland, Centre for Online Health, PAH Telehealth Centre, Princess Alexandra Hospital, Woolloongabba, QLD 4102 Australia

**Keywords:** Telemedicine, Telehealth, Healthcare, Specialists, Rural/remote, Community

## Abstract

**Background:**

Telehealth can offer alternative options for receiving healthcare services in rural locations, improving access and reducing costs associated with traveling for services. However, the full potential of telehealth has not been realised with slow and fragmented uptake. This study describes the awareness, experiences and perceptions of telehealth in an Australian rural community.

**Methods:**

Semi-structured interviews were undertaken with 47 participants from three rural towns in the Darling Downs region of Queensland. Content analysis was used to abstract themes and core concepts from the interviews.

**Results:**

Three participants were healthcare providers who had all previously used telehealth in their clinical practice. Twenty-seven (57 %) participants regularly travelled to access specialist healthcare. While 28 (60 %) participants were aware of telehealth, only six (13 %) had actually used telehealth services; three as patients and three as healthcare providers. Major themes evident included: acceptance of the need to travel; paternalism and empowerment; and trust and misconceptions.

**Conclusions:**

For telehealth initiatives to be successful, there needs to be greater public awareness and understanding of the potential benefits of telehealth. Empowering patients as partners in the delivery of healthcare may be an important factor in the growth of telehealth services.

**Electronic supplementary material:**

The online version of this article (doi:10.1186/s12913-015-1094-7) contains supplementary material, which is available to authorized users.

## Background

**Fig. 1:**
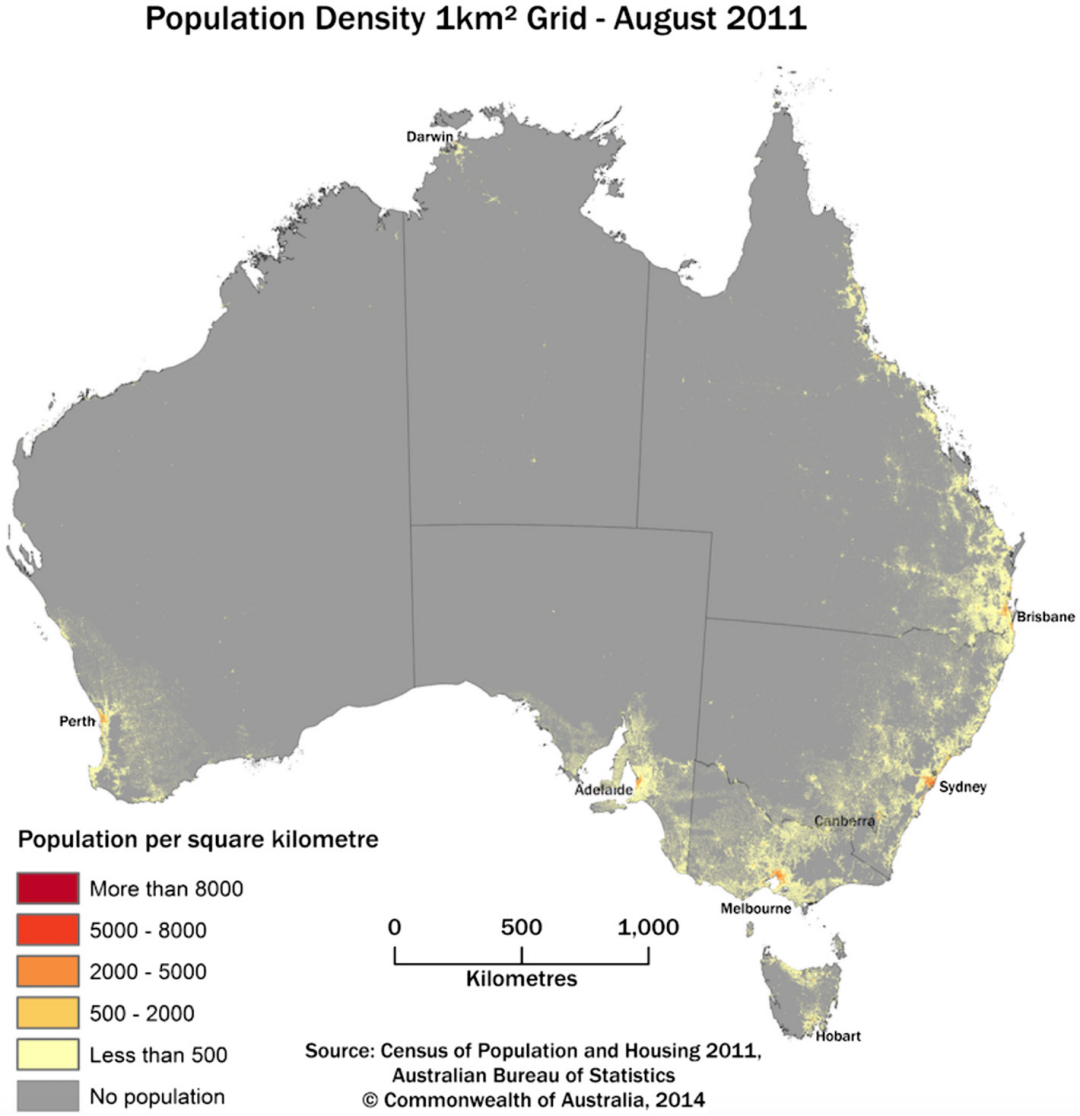
“Australian Population Grid 2011”, Source: Census of Population and Housing 2011, Australia Bureau of Statistics. Copyright Commonwealth of Australia, 2014

Australia is a large continent, with one of the most urbanised populations in the world. Most towns and cities are located within 50 km off the coastal perimeter, with vast unpopulated areas in the middle of the continent (See Fig. 1) [1]. Australia has a low population density (people per square kilometre); in 2014 the Australian population density was just 3 compared with 35 in the United States, 265 in the United Kingdom and 421 in India [2]. Population counts are used to classify Australian locations as either urban or rural. Urban locations are those with a population cluster 10,000 people or more, and rural locations make up the balance [3]. Over the last two decades— similar to many other nations — Australia’s urban population has increased, while the rural population has slowly declined [3]. Unsurprisingly, health outcomes for people living in rural locations of Australia are generally worse than their urban counterparts [4, 5]. National census data estimated life expectancy to be four years shorter, and mortality for people under 65 years of age to be twice as high in rural areas compared with urban areas [6]. This is largely because people living in such locations do not have the same level of access to healthcare. Equity of access to healthcare in rural locations is compromised by geography, time and distance.

The Australian healthcare system attempts to address these inequalities by attracting healthcare practitioners to rural locations with financial incentives or with bonded university scholarships [7]. These incentives facilitate greater access to primary healthcare, but for individuals who require specialised care, access remains challenging.

Queensland is the second largest state of Australia, has a population of around 4.7 million people with approximately 1.6 million (30 %) living in rural locations. Specialist health services across the state are generally available only in large urban centres [8]. Approximately 25–35 % of people living in rural parts of Queensland regularly travel to access specialist healthcare [9]. As well as being inconvenient and disruptive, travelling to access healthcare involves costs for time off work, transport and accommodation [10, 11]. Many of these costs are not measured, or reported, as a cost of consuming healthcare. Additionally, the need to travel for healthcare may result in delays for diagnosis and treatment, which can negatively affect health outcomes.

One solution to address these problems is the use of telehealth to provide healthcare at a distance. Telehealth is defined as ‘the delivery of healthcare services, where distance is a critical factor, by all healthcare professionals using information and communication technologies for the exchange of valid information for diagnosis, treatment and prevention of disease and injuries, research and evaluation, and for the continuing education of healthcare providers, all in the interests of advancing the health of individuals and their communities’ [12]. Communication technologies include videoconferencing, telephony and email; which rely on certain infrastructure and equipment such as the Internet, computer and videoconferencing systems. Telehealth is intended to provide clinical support and improve health outcomes by overcoming geographical barriers through connecting patients and clinicians who are not in the same physical location. Telehealth can deliver specialist services by connecting patients in rural locations with a specialist in another geographic location–and thereby provide timely access, which may improve patient outcomes. Telehealth can also provide education, training and support to rural practitioners thereby increasing their capacity to confidently and capably manage patients in their local area. The usual model for a specialist telehealth consultation is described below and summarised in in Table 2.

Usually, the patient will have previously seen the specialist in-person, or will have a plan for a follow up in-person consultation. Telehealth services are not intended to replace the need for in-person consultations, but to substitute in-person consultations when clinically appropriate. Patients attend a telehealth consultation at their local hospital facility, or from a local general medical practice. Coordination of diagnostic tests results are sent to the specialist prior to the scheduled consultation, or made available for review in real time during the consultation. It is customary and preferable for patients to be supported by medical or nursing staff at the patient end during the telehealth consultation. The supporting clinician is able to undertake physical examination if required and is also responsible for ensuring the patient has understood the outcomes of the consultation. In this way, specialist telehealth consultations can be delivered for a wide variety of clinical reasons such as: pre or post surgical review; endocrinology, oncology, cardiology, neurology etc. review; mental health assessment and intervention, and pre- and post-natal obstetric care.

In Australia, the government and state health services promote telehealth as a solution to the challenge of providing health services across the geographically dispersed country. Indeed there has been a range of government funded financial incentives for specialist consultations, and systemic investments across the country in infrastructure and equipment to facilitate telehealth services [13, 14]. Despite the well-documented benefits of telehealth, (improved patient outcomes, increased access to services, reduced waiting times, reduced travel, education and support) its underuse is consistently reported. The factors that are associated with poor adoption of telehealth into routine practice include clinician acceptance, ethico-legal concerns, organisational readiness, economic restraints and policy directives [15, 16]. Other barriers from the health system perspectives that prevent integration of telehealth services into routine practice are well described and include: administration and coordination; workforce availability; access to equipment and technical support; funding and business models, and telehealth training and education [17, 18]. When compared to the healthcare providers, patients may have a different understanding of the benefits, perceived barriers, and limitations of receiving healthcare through this modality.

As patients may influence the way telehealth services are delivered [19], it is imperative to understand patient awareness and perceptions of telehealth in order to develop services that are acceptable to the community [20]. However, general community perception of the potential of telehealth to address healthcare needs has not been well reported; the literature largely focuses on the perceptions of clinicians and patients that have already experienced telehealth services. There are few reports of community perception of telehealth; Jennet et al. undertook a study in Canada to explore readiness of a rural community for telehealth implementation [21] and Scharwz et al. investigated the readinness of a remote poulation in New South Wales, Australia, to take up e-health innovations [22]. Both studies found the community readiness was moderate, with barriers associated with willingness to try technology solutions. However, both these studies purposively selected participants who were either key informants or patients already using health services, not member of the general public.

In 2010, the Health-e-Regions project was established in the Darling Downs region of Queensland, Australia. The Darling Downs region covers 38,039 km^2^ and consists of small rural towns around a regional centre with a total population of approximately 43,000 people [1, 23]. The project aimed to promote the awareness and use of telehealth services throughout the region, particularly in the towns of Dalby, Chinchilla and Miles. These three towns are located 201, 290 and 336 km respectively from Brisbane, the closest major urban centre. The regional town of Toowoomba, located 84 km from Dalby, also provides limited specialist services. Full details of the Health-e-Regions project have previously been reported [4, 24].

Until the late 90’s, these three towns were predominantly farming communities and all three had experienced rapid growth and development as a result of coal and gas projects. The demographics of the three towns are similar, but compared with the rest of Queensland these towns have a higher proportion of children under the age of 14 years (23 versus 20 %) and older adults over 65 years (15 versus 13 %) and a lower proportion of adults aged 15–64 years (62 versus 67 %). The health statistics for the three towns all differ significantly compared to the rest of Queensland; fertility rates and percentages of vulnerable children are higher, proportions of people on welfare benefits are higher, and rates of risk factors such as smoking, excessive alcohol consumption, and obesity are all higher [4]. Income per capita for each of the three towns is significantly lower compared the major urban centre of Brisbane ($22,000 versus $34,000) [3]. Each town has a public hospital with a range of services, a medical centre and residential aged care facility. Hospital services available in Dalby include day surgery, general medicine, outpatients, emergency and acute care, palliative care and community services. The towns of Chinchilla and Miles are smaller and offer more limited services similar to those available in Dalby except for surgery. Most specialist services are not available locally and patients travel to urban centres (Toowoomba or Brisbane).

The Health-e-Regions project undertook a ‘whole of community’ approach to broadly promote telehealth services in a range of settings over a three-year period (2011–2014). The project facilitated the installation of equipment, education of clinicians, creation of partnerships and networks, and media promotion of telehealth through local radio, newspapers, letterbox drops and community events. The aim of this current study was to explore community awareness, experiences and perceptions of telehealth in the Darling Downs region.

## Methods

The research methods are reported following the Consolidated Criteria for Reporting Qualitative Research (COREQ) [25]. The researchers who undertook this study all have Doctor of Philosophy (PhD) qualifications and are academics in health services research. No researchers had relationships with any of the participants prior to the study. Ethical approval for the study was obtained from the University of Queensland Behavioural and Social Sciences Ethical Review Committee (Ref 2013301482). The study was undertaken between July and August 2014.

### Study design

We used a phenomenological approach to collect field notes and undertake semi-structured interviews with residents of three rural towns — Dalby, Chinchilla and Miles. This approach involves the intention to understand and explore the phenomena from the perspectives of the participants [26]. The interview guide was informed by a literature review in this subject area [27, 28] and piloted on five individuals. Following this process, questions were refined and clarified. Probing questions were used to specifically explore participant’s experiences, awareness and perceptions of access to health services including telehealth (see Additional file 1).

### Sampling

A convenience sample of participants was recruited from local public areas (main street and shopping mall) in all three towns. Participants were required to speak English, be over the age of 18 years and have the capacity to provide informed consent. We attempted to balance the demographic profile of participants and maximise sampling variation by approaching individuals across all age ranges, and ensuring a representative number of males and females were selected [29]. Recruitment continued until a sample of participants was reached that included a wide variety of demographic profiles from all three towns. Interviews were undertaken during normal working hours (9 am–3 pm) over three consecutive weekdays in the three towns. Potential participants were approached face-to-face and asked to participate in one semi-structured interview. All participants were provided with information regarding the study, including the reasons for undertaking the research and provided informed oral consent. Participants were provided with an explanation of telehealth as per Table 2 during the interview. The study authors (NB, LC, AS) conducted the interviews in public places, but in settings such as local parklands, the library, and public resting places where it was unlikely any other person would hear the interview. Field notes were made in each of the three towns to document the settings, atmosphere, events, timing and the researcher’s reflections of the interview processes.

### Data analysis

Interviews were audio recorded and transcribed verbatim. Two researchers (NB, LC) verified the interview transcripts by independently listening to the audio transcript while simultaneously reading the transcript text. Transcripts were not returned to participants. Data were analysed using conventional content analysis approach. Qualitative content analysis is a research method defined by the use of subjective interpretation of the content of data through systematic classification, coding and the identification of themes [30]. The advantage of this method is information is obtained directly from participants without the impositions of pre-conceived categories or theoretical perspectives. This approach involves considering both the manifest and latent content of the data. Manifest content refers to the visible, obvious component, whereas latent content describes the relationship aspects of the data and requires interpretation of the meaning of data [31].

Each interview and field note was considered an individual unit of analysis. Analysis commenced with the reading and re-reading of transcripts to achieve immersion in the data [32]. Discussion between authors was undertaken to examine reflexivity and the possibility of bias and authors’ own influence on the coding. NVivo™ software was used to organise coding of data according to the author’s initial thoughts and impressions. Steps to enhance analytical rigour included multiple coders (NB, LC) to ensure conceptual consistency and inter-rater reliability. For each category and theme, coding rules were defined and verified independently by two researchers (NB, LC). Once all transcripts were coded, data within codes were examined and related concepts grouped into meaningful themes. Constant comparative techniques were used to ensure data coding was consistent with the original data transcripts. Finally, themes were abstracted and developed into core concepts [32]. Exemplars of categories and themes were extracted. The results were then reviewed by all authors and discussed to ensure consensus of findings.

## Results

### Results of sampling

In general, people from all three towns were helpful, friendly, engaging and displayed typical country hospitality and curiosity as to who we were and what we were doing ‘in town’. The majority (70 %) of individuals who were approached agreed to participate in an interview; those who refused (*N* = 20) commonly responded they were too busy to stop. Some individuals indicated they did not want to be approached, e.g. bowing head to avoid eye contact or changing the direction they were walking. These people were not approached. Semi-structured interviews were undertaken with 47 participants; 9 from Chinchilla, 12 from Miles, and 27 from Dalby. Fourteen participants (30 %) were not from the town where the interview was undertaken; most of these participants were travelling to the town to access services (banks, shops, healthcare), or had travelled into the town for work and lived in another location. There were three participants who were healthcare providers included in the sample; two paramedics and one general medical practitioner. The cultural background of the sample is representative of the three towns; 87 % of the population comprise people born in Australia with European ancestry (87 %) and 7 % of the population identify as Indigenous Australians [1]. Characteristics of participants are presented in Table 1.

**Table 1:** Characteristics of participants (*n* = 47)

Characteristics of participants		N	%
Gender			
	Male	26	55
	Female	21	45
Age			
	18–29	10	21
	30–44	12	26
	45–59	9	19
	60–74	10	21
	75+	6	13
Cultural background			
	Caucasian	25	93
	Indigenous Australian	2	6
Presence of a pre-existing health condition requiring specialist care?			
	Yes- self	22	47
	Yes- for partner or family member	5	11
	No	20	43
Self-rated health status (1–10; 1 worst possible health, 10 best possible health)			
	8–10	28	60
	6–7	17	36
	4–5	1	2
	Not stated	1	2
Current employment status			
	Home duties	3	6
	Unemployed	2	4
	Manual labour	10	21
	Sales/Office work	9	19
	Professional	6	13
	Retired	10	21
	Pensioner	7	15
Current Marital status			
	Single	5	11
	Separated, widowed or divorced	4	8
	Married or de-facto relationship	28	60
	Not stated	10	21
Mode of transport			
	Drives self	35	74
	Has family or friend who can drive	6	13
	Relies on public transport	6	13
Has previously heard of telehealth			
	Yes	28	60
	No	19	40
Has previously used telehealth			
	Yes- as a patient	3	6
	Yes –as a healthcare provider	3	6
	No	41	88
Has a positive perception of telehealth			
	Yes	40	85
	No	5	11
	Unsure	2	4
Would use telehealth to access healthcare for themselves/family			
	Yes	39	83
	No	6	13
	Unsure	2	6

The major themes identified were: general acceptance of the need for travel; empowerment and paternalism; and trust and uncertainty. Field notes were used to contextualise the data but did not add further to the themes.

### You live in the bush, you just have to travel

There was a general acceptance within the community that part of living in a rural location meant you had to travel. These sentiments extend beyond the need of travelling for specialist healthcare; respondents reported travelling for basic needs such as groceries, clothing and social events. Travel was stated to be ‘part and parcel of living in the country’, with respondents expressing a ‘we just get on with it’ attitude despite the burden associated with travel. As one respondent declared:

“*Travelling is inconvenient, irritating, annoying and certainly costs a lot of money, but you just have to do it*” (Male #14)

While people living in these rural areas accepted the need for travel, there was the suggestion in several interviews that health service providers may not appreciate or be aware of the efforts patients have undertaken to attend an appointment. Further, they may not be aware of the inequity of access that patients from rural locations experience:

*We always have to travel for healthcare, both for the GP [general medical practitioner] and for a specialist [consultation]. It’s hard for them [healthcare providers] to get their head around the idea we can’t just pop in the next day for follow up or anything like that.* (Female #25)

Participants reported travelling long distances over several days to attend specialist appointments, often at their own expense. The need for travel was not questioned; participants did not perceive there were opportunities to change the system, or that there were any alternative options to access healthcare. There was a sense of simply accepting that was the way things were, these were factors beyond their control. There was the suggestion in several interviews that access to primary and specialist healthcare was becoming more difficult. One participant provided an example of the escalating need for travel as services were increasingly being withdrawn from the town.

*There should be better services. We used to have an operating theatre, and they could deliver babies, but there is nothing now. And there is still the same amount of people there, still the same amount of babies coming. I don’t know why they took all that stuff away from us. Because now every time you get a cut finger you have got to hop on a plane or drive for hours.* (Male #34)

*Sometimes people do need a specialist, but there is only one specialist who comes out here to the hospital once a month. When you are sick, you don’t want to wait, you just want to see the doctor.* (Female #43)

The need to travel for healthcare, coupled with strained local services, limits the control and choice individuals have regarding access to healthcare services. Despite this, 60 % (*N* = 28) participants were happy with the services they had access to, with most stating they had trust in their local doctors and believed they received good primary healthcare.

For patients who are unwell, the irony of travelling for healthcare, when the travel itself has detrimental effects on health, is ever present. Several participants expressed their concern regarding the safety of travelling long distances on the roads, particularly for the aging population. The combination of long distances, road conditions and the stress associated with travelling when unwell, adds further to the burden of illness:

*By the time we get there today [travelling 5 h for specialist appointment], Mum will be flat out exhausted, then when we get home tomorrow, she will be bedridden for two days to get over it [the travel].* (Male #40)

Another participant also explained:

*The nearest centre as far as specialist services is two-and-a-half hours away, that’s a long way for someone who is feeling unwell. (Female #45)*

The main arguments for telehealth are the reduced need for travel and the increase in equity of access to care. However the participants interviewed had limited awareness of these potential benefits for patients, healthcare providers and healthcare systems.

### Awareness of telehealth

While 28 (60 %) participants were in fact aware of telehealth, when asked directly eight participants initially responded that they did not know what telehealth was. However, when the concept was explained, these participants stated they had heard of health services being delivered at a distance using technology. This indicated respondents did not identify with the term ‘telehealth’. One respondent who didn’t know what telehealth was - and who also stated he wouldn’t use telehealth - had actually used a telehealth service (store-and-forward dermatology). This participant had travelled to access a service where photographs were taken of skin lesions that were subsequently sent to a specialist in a different location for review. In a functional telehealth service model, the photographs could have been taken in the participant’s local area and sent to the same specialist for review, eliminating the need to travel.

Participants awareness of telehealth developed through various mediums: the radio; newspaper; television, and through word of mouth. There were 19 participants who had no previous awareness of telehealth. When asked what they thought telehealth was, answers included: a service to buy medications over the Internet; something to do with Medicare; a phone service to discuss health issues.

Once a model of care of telehealth was explained to participants, (see Table 2) they were asked questions regarding their perceptions of telehealth, including if they would use such services themselves.

**Table 2:** Model of telehealth

• Identified need for specialist consultation
• General medical practitioner and patient agree review by telehealth is appropriate
• Specialist appointment organised through general medical practice or local hospital facility
• All required diagnostic tests undertaken in local region and results made available prior to consultation
• Medical practitioner or nurse accompanies patient during specialist consultation

### Empowerment and paternalism

Some participants expressed the desire for greater control over their ability to access health services and recognised the ability of telehealth to facilitate that control and autonomy:

*I would definitely use telehealth. I am pregnant and I have to go in [to town] to see the doctor all the time. If I wanted to see a specialist I would have to travel further. That has affected our decision of where we are having our baby- we don’t want to travel two-and-a-half hours every time we need to see someone.* (Female #25)

The use of telehealth in obstetric care has been generally reported [33]. Specific application in this field includes antenatal, postnatal care, diagnostics and counselling. The idea that telehealth could reduce the need to travel, and that there was a potential alternative method of receiving healthcare was openly received and provoked further discussion and thought:

*Not travelling for five hours when you are sick to see someone, surely that is a better thing. Whether you do it from home or from the nearest hospital or community service it would be a good thing. I think it would be terrific actually.* (Female #45)

*What would be the most important thing to me is that you may be able to avoid the second visits. You know do all your tests and have the face-to-face when you have the results of all the tests, so you could avoid that second visit, coming all that way back in just one week’s time. I actually think it would be great, if your GP [general medical practitioner] or health practitioner was there with you [while you saw the specialist], I think that would be great. The money it would save you, and the time.* (Female #38)

*I wouldn’t have minded doing some appointments by telehealth, but generally they were shoving dye into me and whacking me into a machine. But if it could be done by video it wouldn’t worry me and it would save me ten hours behind the wheel, half a tank of petrol and then some.* (Male#21)

In contemporary healthcare, patient-centred care models include the patient as an active member of the healthcare team. Historically, the patient role has been viewed as a passive role, where the patient is the recipient of healthcare, and not an active member of the healthcare team. Some recipients displayed such an attitude, which prompted the interviewer to ask questions regarding how telehealth services should be promoted. Participants were of the view that telehealth services should be promoted firstly by a general medical practitioner (GP). The GP was viewed as the first point of contact for health issues and the appropriate person to provide information and referrals to specialists. One participant thought there should be a more systemic approach to the promotion of telehealth with information regarding services coming from government and private sectors as well as in general practice.

*Those recommendations should come from the doctors you see, they should say what services are available and they should be saying ‘look try it and if you don’t feel you have got satisfaction from that service then by all means go see someone face-to-face, but they should offer it.* (Female #45)

*I would attack it [telehealth promotion] with everything, because that brings reassurance. If people see it promoted through several different areas then you are not just thinking, ‘oh what’s in it for that particular sector’, it always brings more reassurance. So different parts of society promoting it would be good.* (Female#38)

### Trust and misconceptions

Some participants stated they would want to see a doctor they were already familiar with, and whom they trusted for telehealth to be an acceptable option for the delivery of health services. Ultimately, trust is required for any application of healthcare to be acceptable to patients. Telehealth is no different and patients and practitioners need to be able to trust the health outcomes they are aiming for are achievable through telehealth. No participants voiced opinions that this may not be possible and the few participants who had experienced telehealth all reported positive experiences where healthcare needs were met and the inconvenience of travel was avoided.

Most participants were in favour of receiving services via telehealth and many articulated the benefits usually associated with telehealth. The general consensus of participants was telehealth offered increased access to health services by reducing the need for travel and that particularly for people living in rural areas, telehealth services made practical and economic sense. There were a few participants (*N* = 7, 15 %) who did not think telehealth would be appropriate for them. These participants stated they preferred face-to-face; the inability for physical examination during telehealth consultation was their primary concern. Participants expressed the view that older generations would be less likely to engage with services offered by telehealth.

*“Seeing [a doctor] by the computer! Oh…Oh I am not sure….you can only see a certain bit, you know what I mean? I would be a bit worried on it.”* (Female #43) *and “Well it would work from now on, but us old people, we can’t connect with that modern day thinking. But down the road it will probably work.”* (Male#34)

Other participants had misconceptions regarding the process of telehealth consultations, commonly thinking the patient would need to have equipment in their own home, be computer literate and to organise their own appointment. Once these misconceptions were clarified, and a model of telehealth explained (see Table 2), participants expressed relief that they were not expected to manage the technical aspects of a consultation. This has potentially important implications for home telehealth services.

The few participants that had used telehealth reported positive experiences for both patients and healthcare providers:

*I didn’t have to sit around and wait or anything. By the time I had filled in the hospital forms and walked into the room they had already made the connection. So it was faster, I thought it was great.* (Female #9)

*It’s really good, it works really well. I have a few patients that couldn’t afford transport. It also gives me an opportunity to learn as a junior doctor, If I can I try to talk to my patients rather than just sending them off. So from my point of view, I really like to incorporate that [telehealth] into my practice* (Female #15)

## Discussion

Most studies investigating perceptions of telehealth have focussed on patients or clinicians who have had experience with telehealth consultations. In this study, we sought to understand the broader community awareness and perceptions of telehealth in a rural town of Queensland. We identified that people living in the Darling Downs area of Queensland regularly travelled to access specialist services. It is widely accepted by this population that living in rural areas will necessitate travel for many reasons including health. However, the ability of telehealth to deliver a multitude of quality services challenges the validity of this acceptance. Participants were asked about access to specialist healthcare and their expectations of health services in rural locations. Responses indicated participants perceived they were the recipients of services and that their role as a patient was to accept the advice and services offered to them, regardless of the inconvenience, or burden associated with the required travel. These findings suggest specialist healthcare in this region remains a largely paternalistic system where both patients and healthcare providers accept the system without question*.* While the participants in this study voiced their agreement that the system should be better, and that there should be alternative choices for healthcare available, there was an underlying acceptance that travel was un-avoidable.

A paternalistic health system makes decisions based on what the ‘system’ finds to be in the patient’s best interest, against informed choice, and the patient makes decisions based on the information provided, against interpretive choice [34]. This view of the role of the patient is widely accepted as out-dated; healthcare has evolved considerably over the last 20 years with a patient-centred and shared decision-making approach now regarded as integral to providing a quality health service. Indeed in most parts of urban Australia, patient-centred models of care are well integrated into the whole healthcare system. Telehealth services can be viewed as patient-centric, and as a means of providing the quality and standard of care expected from specialist’s services, but in a location that is practical for the patient. One could argue that telehealth services would be commonplace in a healthcare system that was truly patient-centric. However, telehealth is not the only example of rural health lagging behind in this movement, mental health and palliative care services in rural locations have also reported a slow change to a patient-centred approach [35, 36, 37].

For patients to be able to ask for specialist services by telehealth, they need to know about telehealth and understand the possibilities - and also the limitations - of receiving services through this modality. Additionally, for patients to be able to receive telehealth services, their primary care provider needs to know how to access and refer to such a service. Participants agreed the main benefits of telehealth would be the reduced need for travel and they did not appear to be concerned about the limitations of telehealth or privacy concerns that are reported in the literature [16–18]. Participants alluded to some of the barriers reported in the literature, such as reluctance to change, however this study highlights that lack of community awareness of the availability of telehealth may be another important barrier.

When asked who it should be that promotes telehealth, participants in our study were of the view that the general practitioner was the most important source of information, and they would expect the general practitioner to be the one to offer telehealth services. These responses still conform to the paternalistic view of the roles of the patient and the doctor. It may be the paradigm shift that has occurred through much of society has not reached these rural areas yet and that individuals living in rural areas are more comfortable with a paternalistic model of healthcare; however, the change will still arrive at some future point. The shift away from paternalism and towards self-advocacy and empowerment has occurred across many aspects of society. There has been a societal change with the questioning of authoritative services, a move to more consumerist model with greater accountability and focus on quality [38].

This has affected health services and there is emerging evidence from Australia and overseas that individuals are seeking more control over their healthcare and developing self-advocacy strategies to navigate complex health systems [39, 40]. As self-advocacy regarding healthcare develops, greater flexibility from the system is likely to be required. Recent evidence highlighted diffusion of innovation in healthcare takes as long as 17 years to occur, and that harnessing the efforts of empowered patients and the public as co-producers of wellbeing were one of the most important factors to enable change [41]. These findings highlight the importance of community perceptions and awareness of telehealth as a driver for change across the health system. Our study has highlighted that community awareness of telehealth is not as pervasive as it could be, and as a potentially important driver for telehealth, efforts should be made to inform communities of their options and choices available for healthcare. This may be achieved by greater public promotion and marketing of telehealth as an alternative option for accessing healthcare and should be a priority for health services to increase the demand for telehealth from patients.

### Limitations

Descriptions of new technology tend to be positive or neutral because individuals have a general default tendency towards forming positive attitudes in the absence of negative information (optimism or positivity bias). Negative information is more likely to be present in an actual trial than in a hypothetical scenario [42]. Only six of our participants had experienced telehealth, hence there may have been positivity bias to the number of interviewees that stated they would use telehealth or considered telehealth to be a good idea. As a qualitative study, the findings from this study are not generalizable beyond the sample recruited. Furthermore, as a convenience sample, our participants expressed their own views and experiences; these may not be representative views of the whole community.

## Conclusion

A pragmatic approach was undertaken to obtain information regarding community awareness and perceptions of telehealth in the Darling Downs region of southwest Queensland. Semi-structured interviews and observations were undertaken with local residents and communities to gain an understanding of the perceived benefits, limitations and usefulness of telehealth services. The major themes that developed through the data were: acceptance of the need for travel; empowerment and paternalism; and trust and misconceptions. While there was general awareness of what telehealth was, most people had not experienced telehealth services or considered requesting access to healthcare using this modality. Telehealth offers a promising opportunity to improve the health outcomes for people living outside of metropolitan areas by increasing access to healthcare. Greater community awareness is an important driver for telehealth services and public awareness efforts should focus on increasing community understanding of the options for access to health services including telehealth models of care.

## Additional file

Additional file 1:
**Appendix 1.** (DOCX 67 kb)
