# Supplementary material for: Awareness, experiences and perceptions of telehealth in a rural Queensland community
Source: BMC Health Serv Res. 2015 Sep 28;15:427. doi: 10.1186/s12913-015-1094-7 (PMC4587917; doi:10.1186/s12913-015-1094-7)
Supplement: Additional file 1: — Appendix 1. (DOCX 67 kb) [file 12913_2015_1094_MOESM1_ESM.docx]

# Appendix 1

| **Questions** | **Expanded questions** |
| --- | --- |
| Have you heard about telehealth or telehealth?  Clarify if required. | What do you think telehealth is?  Have you heard or read anything about telehealth?  How did you learn about telehealth? |
| We don’t need to know any specific details about your health, but we are interested in knowing if you or any of your family members ever needed to travel for specialist healthcare? | If yes, explore further:  Can you tell me a bit about your experience of travelling for healthcare?  Time taken for travel?  Time needed off work?  Costs for petrol/parking/accommodation?  Length of appointment?  Did you need any special tests or treatment required?  Did you use the time in Brisbane to do any other things like visit family  Do you think you that appointment could have been done just as well by telehealth ? |
| Have you ever had a telehealth consultation? | If yes, explore further:  How did you feel about the consultation?  How did it compare with a face-to-face consultation?  Were you comfortable with the consultation?  Were you confident the consultation was done just as well as if you saw the doctor in person?  Did you understand the advice you were given?  Will you have further consultations this way?  Do you think telehealth consultations saved you time or money?  Was this a convenient way to see a doctor?  Were there any downsides with seeing a doctor this way? |
| How would it help if you saw a doctor via telehealth instead of travelling? | Would it save you time or money?  Would it be convenient way to see a doctor?  Would you have any concerns with seeing doctor this way? |
